# AFTER THE STORM: ASSOCIATIONS AMONG HOPE AND POSTTRAUMATIC GROWTH IN YOUNGER AND OLDER ADULTS

**DOI:** 10.1093/geroni/igae098.2368

**Published:** 2024-12-31

**Authors:** Piper Bordes, Kimberly Belalcazar, Katie Cherry

**Affiliations:** Louisiana State University, Baton Rouge, Louisiana, United States; Louisiana State University, Baton Rouge, Louisiana, United States; Louisiana State University, Baton Rouge, Louisiana, United States

## Abstract

Hurricane Ida devastated south Louisiana on August 29, 2021 having psychosocial consequences for those directly and indirectly impacted by this environmental event. The present research is part of a larger study on positive factors that enhance long-term disaster recovery. In this study, we factorially crossed age (younger, older) and disaster exposure (indirect, direct) in order to examine their influence on post-traumatic growth with greater precision than previous work in a disaster context. Participants were sampled from three parishes (counties) where Ida damage was more severe and two parishes which were farther inland with less damage. All were tested in person or virtually via Zoom platform in a single session where they responded to several individual difference measures, a structured storm questionnaire and survey measures of state hope and post traumatic growth. Results indicated that state hope scores were comparable across the age and exposure groups. Older adults had greater post-traumatic growth than did their younger counterparts, although the two exposure groups did not differ. Correlation analyses revealed that hurricane stressors were negatively associated with hope and positively associated with fear during heavy storms and worry about damage to home since Hurricane Ida. These findings support the hypothesis that older age may be a protective factor for well-being after exposure to a devastating hurricane. Implications of these data for understanding the beneficial role of positive psychological factors in long-term hurricane recovery are discussed.

